# Different Effects of Leucine Supplementation and/or Exercise on Systemic Insulin Sensitivity in Mice

**DOI:** 10.3389/fendo.2021.651303

**Published:** 2021-05-12

**Authors:** Xiaofan Jiang, Yuwei Zhang, Weichao Hu, Yuxiu Liang, Liang Zheng, Juan Zheng, Baozhen Wang, Xin Guo

**Affiliations:** ^1^ Department of Nutrition and Food Hygiene, School of Public Health, Cheeloo College of Medicine, Shandong University, Jinan, China; ^2^ Department of Endocrinology, Union Hospital, Tongji Medical College, Huazhong University of Science and Technology, Wuhan, China; ^3^ Hubei Provincial Clinical Research Center for Diabetes and Metabolic Disorders, Wuhan, China

**Keywords:** exercise, leucine, inflammation, insulin resistance, high fat diet, metabolism

## Abstract

**Objective:**

Obesity-related diseases such as diabetes, hypertension, dyslipidemia, and cardiovascular diseases have increased due to the obesity epidemic. Early intervention for obesity through lifestyle and nutrition plays an important role in preventing obesity-related diseases. Therefore, the purpose of this study is to explore the role of leucine and exercise in adiposity, systemic insulin resistance, and inflammation to provide theoretical and guiding basis for the early prevention and treatment of obesity.

**Methods:**

C57BL/6J male mice were randomly divided into HFD or LFD-fed mice group. After 9 weeks, glucose tolerance test (GTT) was performed to detect their systemic insulin sensitivity. Starting from week 10, mice were divided into eight groups and treated with moderate exercise or/and 1.5% leucine. At week 13, systemic insulin sensitivity was detected by GTT. At week 14, mice were dissected to analyze adiposity and inflammation.

**Results:**

In LFD mice, exercise significantly increased systemic insulin sensitivity by increasing GLUT4 expression in the muscle and decreasing adiposity through increasing AMPK phosphorylation in adipose tissue. In HFD mice, the simultaneous intervention of exercise and leucine increases systemic insulin sensitivity by reducing liver and adipose tissue inflammation *via* decreasing NF-*κ*B p65 phosphorylation, and increasing the expression of adiponectin in adipose tissue.

**Conclusion:**

There are different mechanisms underlying the effects of exercise and leucine on insulin resistance and inflammation in LFD-fed mice or HFD-fed mice.

## Introduction

Obesity is one of the most serious health problems in many countries. Due to the prevalence of obesity beginning in childhood, the incidence of metabolic diseases such as diabetes, non-alcoholic fatty liver disease (NAFLD), cardiovascular disease (CVD), and some cancer will continue to increase (1). Therefore, reducing the risk of obesity, especially the early intervention of obesity, is the top priority of public health. The root of metabolic diseases includes the increased energy intake, especially high fat and high refined carbohydrate consumption, and sedentary lifestyle (2, 3). More and more evidence show that inflammation is the main cause of over-nutrition induced insulin resistance and type 2 diabetes (4). Inflammation caused by over-nutrition often shows chronic inflammation, which is different from inflammation caused by infection (5, 6). In the prevention and treatment of metabolic diseases, it is very important to suppress inflammation, improve insulin sensitivity, and improve the balance of systemic metabolism. Many pieces of evidence show that inflammation in adipose tissue and liver plays an important role in the pathogenesis of type 2 diabetes. Mechanistically speaking, over-nutrition can activate the inflammatory signal pathway and release a large number of inflammatory cytokines to interfere with signal transduction, thus inducing adipose tissue (7) and liver (8) inflammation, which is a major reason for systemic insulin resistance. On the other hand, insulin resistance aggravates adipose tissue (9) and liver (10) inflammation. It leads to a vicious spiral and promotes the development of type 2 diabetes.

As we all know, keeping active exercise can reduce the risk of insulin resistance in adults. Other studies have shown that it is also applicable to adolescents. A study on 108 obese adolescents in Taiwan, China (11) found that continuous 12 weeks exercise can improve insulin sensitivity and maintain glucose homeostasis. In human, exercise can improve glucose and lipid metabolism, improve insulin sensitivity, and reduce systemic insulin resistance (12). Exercise reduces chronic inflammation in muscle, adipose tissue, and endothelial cells including reducing leukocyte adhesion and cytokine production. In the immune system, exercise reduces pro-inflammatory cells and reduces the production of pro-inflammatory cytokines (13). In muscle cells, exercise activates 5′AMP-activated protein kinase (AMPK) pathway, facilitating the translocation of glucose transporter 4 (GLUT4) from cytosol to cell membrane to uptake glucose. In addition, exercise also increases mitogenesis *via* the mitogen-activated protein kinase (MAPK) signaling pathways (14). Exercise is reported to increases PPAR*α*-mediated *β*-oxidation, reduce intrahepatic lipid content and hepatic insulin resistance in patients with type 2 diabetes (15). In adipose tissue, exercise increases the level of GLUT4 (16), uncoupling protein 1 (UCP1), and *β*-oxidation (17). Lines of evidence show that leucine not only acts as a nutrient, but also acts as a regulator (18), and supplementation of leucine can increase insulin sensitivity in the muscle and liver. Leucine can increase the mRNA level of uncoupling protein 3 (UCP3) in oxidative glycolysis of skeletal muscle. UCP3 abundance is closely related to skeletal muscle glucose metabolism, and UCP3 affects glucose uptake through translocation of GLUT4 (19). In hepatic cells, leucine increases AMPK phosphorylation, glucose uptake, and triglyceride (TG) accumulation. Myostatin is involved in leucine-mediated glucose uptake through AMPK activation (20). Leucine also promotes glucose-stimulated insulin secretion *via* increasing the levels of glucokinase and ATP synthase in pancreatic *β*-cells (21). In apoE null mice, leucine supplementation can effectively reduce atherosclerosis by improving plasma lipid profile and reducing systemic inflammation (22).

As the prevalence of overweight and obesity in children and adolescents has increased significantly, metabolic diseases such as dyslipidemia, hypertension, type 2 diabetes, and cardiovascular disease have also increased significantly. Early intervention of obesity plays an important role in preventing metabolic diseases in later life. Exercise and leucine intervention may improve obesity and related metabolic diseases (23). However, the effects of leucine supplementation and exercise on insulin resistance and inflammation in early obesity are unknown. Here, we fed mice with high fat diet (HFD) for 9 weeks to get the onset of obesity, then conducted short-term intervention of exercise or/and leucine for 4 weeks. Therefore, the purpose of this study is to discuss the role and possible mechanism of leucine and exercise in adiposity, systemic insulin resistance, and inflammation, and to provide a theoretical basis to guide the prevention and treatment of early obesity.

## Materials and Methods

### Animal Experiments

C57BL/6J male mice aged 5–6 weeks were raised in SPF animal laboratory with controllable temperature and humidity. After one week of adaptation, they were randomly divided into HFD mice group (D12492) or low-fat diet fed (LFD) mice group (MD12450B) according to their body weight. They could drink water freely, and their body weight and food consumption were recorded every week. The fat content of HFD and LFD is 60% and 10%, respectively, in order to provide energy from different fat sources to build models. After feeding for 9 weeks, glucose tolerance test (GTT) was performed. From the 10th week, mice were treated with moderate-intensity exercise or/and leucine and were divided into low-fat feeding group (LFD group), low-fat feeding and exercise groups (LFD + Ex group), low-fat feeding and leucine intervention group (LFD + Leu group), low-fat feeding and combination of exercise and leucine intervention group (LFD + Ex + Leu group), high-fat feeding group (HFD group), high-fat feeding and exercise group (HFD + Ex group), high-fat feeding and leucine intervention group (HFD + Leu group), high-fat feeding and combination of exercise and leucine intervention group (HFD + Ex + Leu group). In the 13th week, GTT was performed to evaluate glucose clearance. In the 14th week, mice were fasting overnight and sacrificed, blood was collected, and tissues were weighed and collected. All procedures were approved by the Institutional Animal Care and Use Committee at Shandong University and performed in conformance with the guide.

### Exercise Intervention

Before the intervention, mice adapted to the smell of running equipment (ZH-PT animal experimental treadmill, # ZH0084.3) and training room for three consecutive days including 10 min visual familiarity and 10 min slow walking on treadmill, to reducing psychological pressure. During the experiment, the runway speed was increased by grading, so that the mice could adapt to the challenge of 10 m/min load, and run for 30 min every other day at this fixed speed. If the low-speed behavior exceeds the limited time in the process, a slight electric shock will be produced at the back of the treadmill, which will not damage the animals, ensuring the accuracy of the research, the operation lasts for 4 weeks.

### Leucine Intervention

The concentration of leucine is 1.5%, which is directly added to drinking water; the operation lasts for 4 weeks.

### Glucose Tolerance Test

Glucose tolerance tests were performed as previously described (24). Before the experiment, mice starved overnight, and the weight was measured the next day. D-glucose (2 g/kg) (Sigma, #47829) was injected intraperitoneally. Blood glucose concentration from tail vein was detected 0 min before injection and 30, 60, 90, and 120 min after injection by blood glucose meter (Roche ACCU-CHEK Performa).

### Tissues Collection

Mice were fasted overnight. The blood of mice was collected by cardiac puncture using 1 ml syringe. After taking blood, the mice were sacrificed by dislocation of cervical vertebra according to humanitarian principle. Samples of whole liver, (visceral, subcutaneous, brown) adipose tissue, and muscle were weighed and collected. Some tissue samples were either fixed and embedded for histological analyses or frozen in liquid nitrogen and then stored at –80°C for further analyses.

### HOMA-IR Calculation

Fasting bloods were collected and centrifuged at 12,000 rpm and 4°C for 5 min. Serums were collected to measure fasting blood glucose and insulin. Fasting blood glucose was measured by glucose assay kit (glucose oxidase method) (Jingmei, #F006-1-1). Insulin level was measured by mouse insulin ELISA kit (Invitrogen, #EMINS 96 test). The formula of HOMA-IR was that the level of fasting blood glucose (mmol/L) was multiplied by the level of fasting insulin (μU/ml), then divided by 22.5.

### Western Blot Analysis

Lysates were prepared from frozen tissue samples. Western blots were conducted. The levels of phospho-NF-*κ*B p65, NF-*κ*B p65 (Cell Signaling Technology #8214), phospho-AMPK (Cell Signaling Technology #2535), AMPK (Cell Signaling Technology #5831), GLUT4 (Cell Signaling Technology #2213), and tubulin (Cell Signaling Technology #2125) were analyzed as described (25, 26).

### Immunohistochemistry and Hematoxylin–Eosin Staining

Liver, muscle, and adipose tissues were fixed in neutral formalin for 72 h and made into slices with a thickness of 5 μm for HE staining and 8 μm for IHC. After that, HE staining or IHC was performed and observed under microscope. For IHC, the antibody was F4/80 (1:100) (Cell Signaling Technology, #70076) (27, 28).

### Real-Time Quantitative Polymerase Chain Reaction

Total RNA was extracted from ground liver, adipose tissue, and muscle with TRIzol and purified by miRNeasy Micro Kit (Qiagen. #217084). cDNA was obtained by reverse transcription from total RNA using SuperScript IV Reverse Transcriptase (Invitrogen, # 18090010), and mRNA expression levels were analyzed by Roche LC480 fluorescence quantitative PCR (#28424) using Platinum™ Taq DNA Polymerase (Invitrogen, # 15966005) (29). In liver, the expressions of acetyl -CoA carboxylase1 (ACC1), fatty acid synthase (FAS), carnitine palmitoyltransferase 1A (CPT1A), phosphoenolpyruvate carboxykinase (PEPCK), glucose-6-phosphatase (G6Pase), glucokinase (GK), and tumor necrosis factor α (TNFα**)** were measured. In adipose tissue; the levels of adiponectin and glucose transporters 4 (GLUT4) were measured. In the muscle, the levels of GLUT4 were determined. Primers of those genes were shown as in **Table 1**.

**Table 1:** Primer sequences for metabolic related genes.

Gene	Primer sequence
Acetyl -CoA carboxylase 1, ACC1	Forward:GGGAACATCCCCACGCTAAA
	Reverse:GAAAGAGACCATTCCGCCCA
Adiponectin	Forward:CCCTCCACCCAAGGGAACT
	Reverse:TTCAGCTCCTGTCATTCCAACA
Carnitine palmitoyltransferase 1A, CPT1A	Forward:TGGGGAGGAGACAGACACCA
	Reverse:CAGCCTCCCGTCATGGTAGA
Fatty acid synthase, FAS	Forward:TGAACCTTGACAGGGCAACC
	Reverse:ATGGTAGAGTTGGCGAAGCC
Glucokinase, GK	Forward:CCCTGTAAGGCACGAAGACA
	Reverse:AGTCCCACGATGTTGTTCCC
Glucose-6-phosphatase, G6Pase	Forward:TGGGCATCAATCTCCTCTGG
	Reverse:AATACGGGCGTTGTCCAAAC
Glucose transporters 4, GLUT4	Forward:TTCACGTTGGTCTCGGTGCT
	Reverse:GGCCACGATGGAGACATAGC
Phosphoenolpyruvate carboxykinase, PEPCK	Forward:ATCTTTGGTGGCCGTAGACC
	Reverse:TTTGCCGAAGTTGTAGCCGA
Tumor necrosis factor α, TNFα	Forward: CACAGAAAGCATGATCCGCGACGT
	Reverse:CGGCAGAGAGGAGGTTGACTTTCT

### Measurement of Liver Triglyceride Level

Frozen livers were extracted and measured for triglyceride levels using a triglyceride (TG) enzymatic assay kit from Applygen.

### Statistical Analysis

Numeric data are presented as means ± standard error of the mean (SEM). Statistical significance was assessed by unpaired, two-tailed ANOVA or Student**’**s t test. Differences were considered significant at the two-tailed p **<**0.05.

## Results

### Effects of HFD on Body Weight, Fat Content, Liver Weight, and Systemic Insulin Sensitivity

At the beginning of the experiment, there was no difference in body weight between the two groups, but the body weight increased during feeding. After 5 weeks, the animals on HFD gained more weight than those on LFD, and with the increase of feeding weeks, the difference between the two groups gradually increased (**Supplementary Data** and **Figure S1A**). Compared with the weight of white adipose tissue of mice on HFD and LFD, adiposity including epididymal fat, perirenal fat, and mesenteric fat of mice fed with HFD increased significantly (**Supplementary Data** and **Figures S1B, C**). In addition, the liver weight of mice fed with HFD increased obviously (**Supplementary Data** and **Figure S1B**). The results indicated that HFD increased the body weight and fat deposition in mice. According to GTT experiment, the fasting baseline blood glucose level of mice fed with HFD was higher than that of LFD, and the difference of glucose level at 30 and 60 min was statistically significant (**Supplementary Data** and **Figure S1D**). For the area under curve (AUC) of GTT, HFD group had a significant difference compared with the LFD group (**Supplementary Data** and **Figure S1E**), indicating that high fat feeding impaired glucose metabolism.

### Effects of Exercise and/or Leucine on Body Weight, Organ Weights, and Systemic Insulin Sensitivity Under LFD

**Figure 1:**
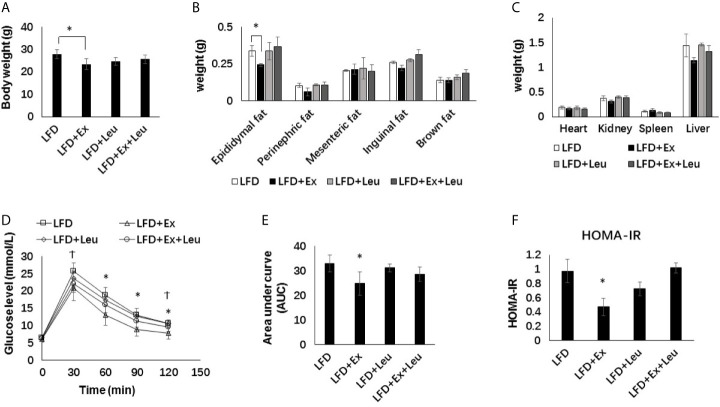
Exercise intervention reduced fat content and improved insulin sensitivity under LFD. C57BL/6J male mice of 5–6 weeks were fed with LFD. Leucine with a concentration of 1.5% and/or moderate intensity exercise was added in the 10th week and administrated for 4 weeks. **(A)** Changes in body weight. **(B)** Changes in fat content. **(C)** Changes in organ weight. **(D)** Glucose tolerance test (GTT). Mice were fasting overnight; D-glucose (2 g/kg) was injected intraperitoneally. Blood glucose concentration was detected 0 min before injection and 30, 60, 90, and 120 min after injection. **(E)** Area under curve of GTT. **(F)** HOMA-IR. Calculated based on fasting glucose level (mmol/L) and fasting insulin level (μU/ml). The data are mean ± s.d. (error bars), n = 6 mice per group. *p < 0.05 LFD *versus* LFD + Ex; †, p < 0.05 LFD *versus* LFD + Ex + Leu.

When mice fed with LFD were compared to the LFD control group, exercise intervention reduced body weight (**Figure 1A**) and epididymal fat (**Figure 1B**). LFD + Leu and LFD + Ex + Leu groups did not show significant weight loss and change of fat content. Other organ weights had no significant statistical difference among groups (**Figure 1C**). Compared with the LFD control group, the LFD + Ex group and LFD + Ex + Leu group enhanced glucose utilization (**Figure 1D**). For the area under curve (AUC) of GTT, the LFD + Ex group had a significant difference compared with the LFD control group (**Figure 1E**). Moreover, HOMA-IR was lower in the LFD + Ex group (**Figure 1F**) compared to other groups, indicating that exercise only can increase insulin sensitivity. Taken together, exercise reduced body weight and fat adiposity, as well as increased glucose clearance and insulin sensitivity in mice during LFD.

### Effects of Exercise and/or Leucine on Body Weight, Organ Weights, and Systemic Insulin Sensitivity Under HFD

**Figure 2:**
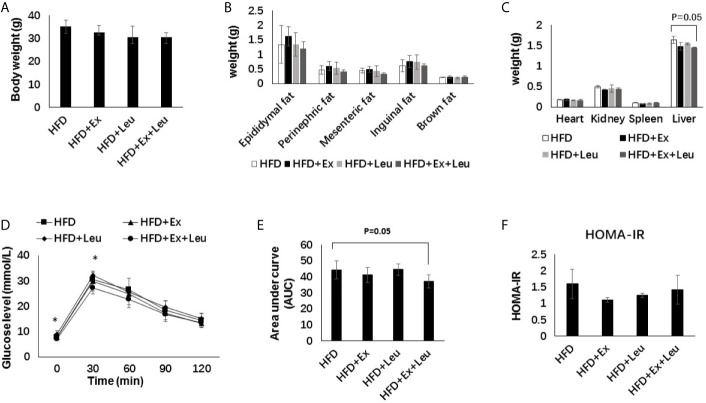
The intervention of exercise and leucine improve insulin sensitivity under HFD. C57BL/6J male mice of 5–6 weeks were fed with HFD. Leucine with a concentration of 1.5% and/or moderate intensity exercise was added in the 10th week and administrated for 4 weeks. **(A)** Changes in body weight. **(B)** Changes in fat content. **(C)** Changes in organ weight. **(D)** Glucose tolerance test. Mice were fasting overnight; D-glucose (2 g/kg) was injected intraperitoneally. Blood glucose concentration was detected 0 min before injection and 30, 60, 90, and 120 min after injection. **(E)** Area under curve of GTT. **(F)** HOMA-IR. Calculated based on fasting glucose level (mmol/L) and fasting insulin level (μU/ml). The data are mean ± s.d. (error bars), n = 6 mice per group. *p < 0.05 HFD *versus* HFD + Ex + Leu.

When fed mice with HFD, the body weight and fat content between the exercise and/or leucine groups did not change significantly (**Figures 2A, B**), and there was no significant statistical difference in tissue weight (**Figure 2C**). It is worth noting that the liver weight of the HFD + Ex + Leu group slightly decreased, and if the experimental time was prolonged, there might be a more obvious difference (**Figure 2C**). Compared with the HFD control group, the HFD + Ex + Leu group had enhanced glucose clearance, which indicated that a combination intervention of exercise and leucine could reduce glucose intolerance induced by HFD, but only leucine treatment or exercise intervention had no effect on reducing glucose intolerance induced by HFD (**Figure 2D**). For the area under curve (AUC) of GTT, the HFD + Ex + Leu group exhibited a lower AUC compared to the HFD control group; however, it may need an intervention for a longer time to show a more obvious difference (**Figure 2E**). HOMA-IR did not show differences among groups (**Figure 2F**). The results suggested that short-term intervention of exercise and leucine had a trend on reducing insulin resistance induced by HFD. A long term intervention is needed to see a significant increase in insulin sensitivity in HFD-fed mice.

### Effects of Exercise and/or Leucine on Adiposity and AMPK Activation in Adipose Tissue

**Figure 3:**
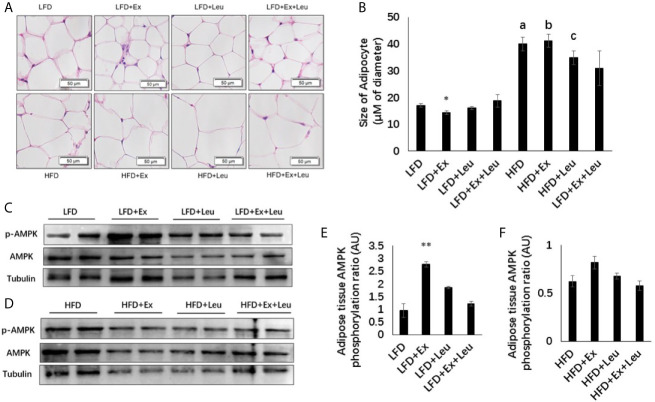
Exercise reduced the size of adipocytes and enhanced AMPK phosphorylation under LFD. C57BL/6J male mice of 5–6 weeks were fed with LFD or HFD. Leucine with a concentration of 1.5% and/or moderate intensity exercise was added in the 10th week and administrated for 4 weeks. **(A)** Adipose tissue histology. The sections of adipose tissue were stained with hematoxylin and eosin. **(B)** Quantification of the adipocyte size. For **(C, D)**, the levels and phosphorylation states of AMPK in the adipose tissue were determined using western blots. For **(E, F)**, quantification of AMPK phosphorylation (arbitrary units) for **(C, D)**, respectively. The data are mean ± s.d. (error bars), n = 6 mice per group. *p < 0.05; **p < 0.01, LFD *versus* LFD+Ex; a, p < 0.01, LFD *versus* HFD; b, p < 0.01, LFD + Ex *versus* HFD + Ex; c, p<0.05, LFD + Leu *versus* HFD + Leu.

Compared with the LFD group, HFD significantly increased the size of adipocytes (**Figures 3A, B**). The sizes of adipocytes in the LFD + Ex group were significantly reduced compared with the LFD control group (**Figures 3A, B**), indicating that exercise reduced adiposity in the adipose tissue. The reduction of adiposity in the adipose tissue may be caused by decreasing fat synthesis and/or increasing fatty acid oxidation. Activation of AMPK results in phosphorylation and inactivation of ACC, a key enzyme regulating fatty acid synthesis to reduce adiposity (30). Indeed, in LFD-fed mice, exercise significantly increased the phosphorylation of AMPK compared to control mice in the adipose tissue (**Figures 3C, E**). In HFD-fed mice, there was no obvious difference on the phosphorylation of AMPK among different groups in the adipose tissue (**Figures 3D, F**). In brief, exercise reduced fat accumulation in the adipose tissue during LFD feeding but was not enough to reduce adiposity during HFD feeding. Exercise might decrease adipose tissue adiposity *via* AMPK activation during LFD feeding.

### Effects of Exercise and/or Leucine on Inflammation and Expression of Metabolic Related Genes in Adipose Tissue

**Figure 4:**
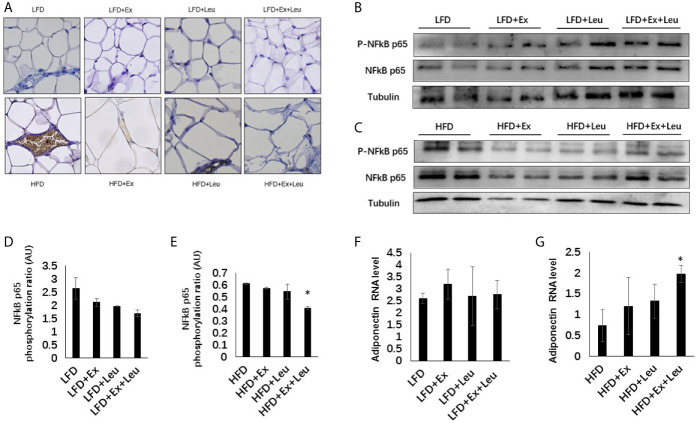
Intervention of exercise and leucine enhanced adiponectin secretion and reduced adipose tissue inflammation under HFD. C57BL/6J male mice of 5–6 weeks were fed with LFD or HFD. Leucine with a concentration of 1.5% and/or moderate intensity exercise was added in the 10th week and administrated for 4 weeks. **(A)** Adipose tissue immunohistochemistry. The sections of adipose tissue were immunostained for F4/80. For **(B, C)**, the levels and phosphorylation states of NF-*κ*B p65 were determined using western blots. For **(D, E)**, quantification of inflammatory signaling (arbitrary units) for **(B, C**), respectively. For **(F, G)**, changes in adipose mRNA level of adiponectin by RT-PCR under LFD and HFD, respectively. The data are mean ± s.d. (error bars), n = 6 mice per group. *p < 0.05, HFD *versus* HFD + Ex + Leu.

HFD significantly increased macrophage infiltration in adipose tissue (**Figure 4A**). When HFD-fed mice was administrated with exercise, leucine, or both leucine and exercise, macrophage infiltration was all decreased; however, macrophage almost disappeared in HFD-fed mice treated with both leucine and exercise (**Figure 4A**). In addition, the inflammatory response induced by HFD was decreased, which showed by the decreased level of Phospho-NF-κB p65 in combination with leucine and exercise treatment (**Figures 4C, E**), while in mice fed with LFD, the inflammatory response did not show obvious difference among groups (**Figures 4B, D**). In adipose tissue, exercise had a trend to increase the expression of GLUT4 (data are not shown here) and adiponectin (**Figure 4F**), but the effect is not obvious during LFD feeding. When mice were fed with HFD, the combined intervention of leucine and exercise increased the mRNA level of adiponectin (**Figure 4G**), but did not increase the mRNA level of GLUT4 (data are not shown here). The result indicated that leucine and exercise might reduce insulin resistance through decreasing the inflammatory response and increasing the level of adiponectin in adipose tissue in HFD-fed mice.

### Effects of Exercise and/or Leucine on Adiposity, Inflammation, and Expression of Metabolic Related Genes in Liver

**Figure 5:**
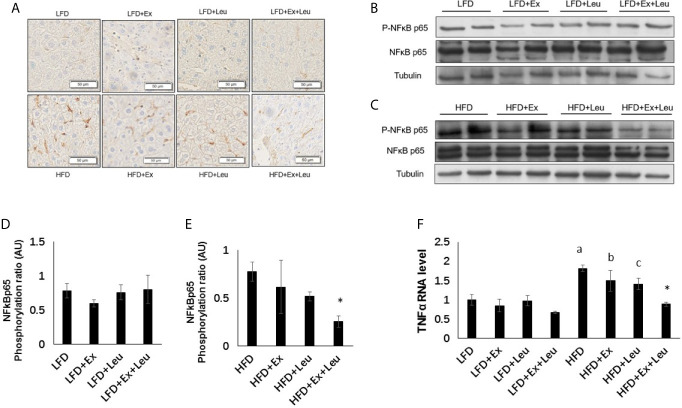
The intervention of exercise and leucine reduced liver inflammation under HFD. C57BL/6J male mice of 5–6 weeks were fed with LFD or HFD. Leucine with a concentration of 1.5% and/or moderate intensity exercise was added in the 10th week and administrated for 4 weeks. **(A)** Macrophage infiltration in the liver. The sections of liver were immunostained for F4/80. For **(B, C)**, the levels and phosphorylation states of NF-*κ*B p65 were determined using western blots. For **(D, E)**, quantification of inflammatory signaling (arbitrary units) for **(B, C)**, respectively. **(F)** Changes in liver mRNA level of TNFα under LFD or HFD using RT-PCR. The data are mean ± s.d. (error bars), n = 6 mice per group. *p < 0.05, HFD *versus* HFD + Ex + Leu; a, p < 0.01, LFD *versus* HFD; b, p < 0.05, LFD + Ex *versus* HFD + Ex; c, p = 0.053, LFD + Leu *versus* HFD + Leu.

The liver plays an important role in metabolism and interacts with all tissues and organs (31). Histological examination showed that compared with the LFD group, HFD increased liver fat accumulation (**Supplementary data** and **Figures S2A, B**), but the treatment of exercise and/or leucine had not shown effects on elimination of adiposity in the liver (**Supplementary Data** and **Figures S2A, B**), which was consistent with the results of tissue weight. Immunohistochemistry results, which blotted with F4/80 to detect macrophage infiltration in the liver, showed that compared with the LFD group, HFD increased liver inflammation (**Figure 5A**). The combination treatment of exercise and leucine significantly alleviated the inflammation in the liver caused by HFD (**Figure 5A**). In mice fed with LFD, exercise had a trend to reduce the level of Phospho-NF-*κ*B p65 (**Figures 5B, D**). When HFD-fed mice were administrated with leucine and exercise, the inflammatory response induced by HFD was decreased, which was shown by the decreased the level of Phospho-NF-*κ*B p65 (**Figures 5C, E**). The mRNA levels of TNFα were obviously increased in the HFD-fed groups. The intervention of leucine and exercise significantly reduced the levels of TNFα induced by HFD (**Figure 5F**), which was consistent with the result for Phospho-NF-*κ*B p65. The expression of glucose metabolism and gluconeogenesis related genes, such as GK, G6Pase, and PEPCK, and lipid metabolism related genes ACC1, FAS, and CPT1A in the liver were detected, and it was not found that leucine or/and exercise had an effect on the RNA expression of these genes during LFD or HFD feeding (data are not shown here). The results suggested that leucine and exercise might reduce insulin resistance through decreasing the inflammatory response induced by HFD in the liver.

### Effects of Exercise and/or Leucine on Adiposity, Inflammation and Expression of GLUT4 in Muscle

**Figure 6:**
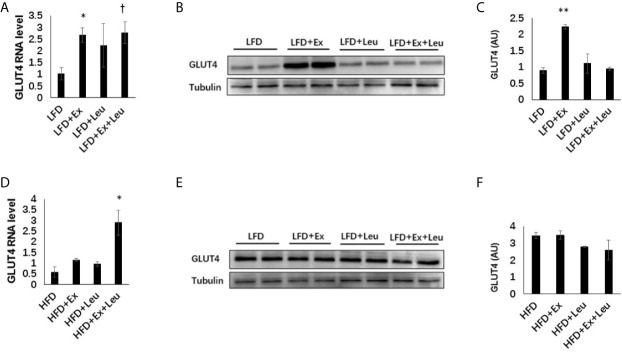
Exercise increased the level of GLUT4 in muscle under LFD. C57BL/6J male mice of 5–6 weeks were fed with LFD or HFD. Leucine with a concentration of 1.5% and/or moderate intensity exercise was added in the 10th week and administrated for 4 weeks. **(A)** Changes in muscle mRNA level of GLUT4 under LFD using RT-PCR. **(B)** The levels of GLUT4 were determined using western blots. **(C)** Quantification of GLUT4 expression (arbitrary units) for **(B)**. The data are mean ± s.d. (error bars), n = 6 mice per group. *p < 0.05, **p < 0.01, LFD *versus* LFD + Ex; †, P < 0.05, LFD *versus* LFD + Ex + Leu. **(D)** Changes in muscle mRNA level of GLUT4 under HFD using RT-PCR. **(E)** The levels of GLUT4 were determined using western blots. **(F)** Quantification of GLUT4 expression (arbitrary units) for **(E)**. The data are mean ± s.d. (error bars), n = 4–6 mice per group. *p < 0.05, HFD *versus* HFD + Ex + Leu.

Muscle TG levels showed that HFD fed mice increased adiposity in the muscle (**Supplementary Data, Figure S3A**) compared to LFD fed mice. Immunohistochemistry (data are not shown here) did not show any difference among the different groups either in LFD or HFD feeding in the muscle. Moreover, the phosphorylation NF-*κ*B p65 did not exhibit obvious differences among groups either in LFD-fed mice or HFD-fed mice (**Supplementary Data** and **Figure S3B**). The expression of GLUT4 in the muscle was detected by RT-PCR and western blot. The results showed that exercise significantly increased the expression of GLUT4 in the muscle when mice were fed with LFD (**Figures 6A–C**). In the presence of leucine, exercise could also significantly increase the mRNA level of GLUT4, but not the protein level of GLUT4. Only leucine treatment could not effectively increase the expression of GLUT4 in the muscle (**Figures 6A–C**). When mice were fed with HFD, in the presence of leucine, the mRNA level of GLUT4 was increased by exercising intervention with statistical significance (**Figure 6D**), but the protein level did not change (**Figures 6E, F**). Taken together, exercise and/or leucine treatment did not change the inflammatory status in the muscle; however, exercise alone significantly increased the expression of GLUT4 during LFD feeding.

## Discussion

At present, although the world has carried out continuous research and public policy efforts, the prevalence of obesity is still the main public health threat, so it is urgent to found new strategies to prevent and treat obesity (32). Studies showed that the nutritional imbalance in children and adolescents will increase the risk of obesity in the future (33, 34). Therefore, this study is devoted to early obesity, which established obesity model by 9 weeks HFD feeding. We observed that body weight had a statistical difference between LFD and HFD starting from the sixth week of HFD feeding (**Supplementary Data, Figure S1A**). Adiposity and glucose intolerance due to HFD feeding were significantly increased at 9th week (**Supplementary Data, Figures S1B–E**). These results indicated that mice fed with HFD for 9 weeks obviously exhibited obese phenotype. During HFD feeding, we treated mice with exercise or/and leucine for 4 weeks. Therefore, the total HFD feeding was 13 weeks. At the end of the study, we observed HFD significantly increased adiposity in the adipose tissue (**Figures 3A, B**), liver (**Supplementary Data** and **Figure S2**), and muscle (**Supplementary Data** and **Figure S3A**). In addition, HFD significantly increased adipose tissue (**Figure 4A**) and liver inflammation (**Figures 5A, F**). Therefore, this study mainly investigated the short-term effects of exercise and/or leucine on the changes in metabolic characterization in mice under LFD-fed or HFD-fed early obesity. Our results show that: 1) Exercise significantly increased systemic insulin sensitivity in LFD-fed mice; however, leucine intervention did not show significant effects on metabolic change in LFD-fed mice. Further research indicated that exercise could increase systemic insulin sensitivity by reducing adiposity and increasing AMPK phosphorylation in adipose tissue, as well as increasing GLUT4 expression in muscle. 2) In HFD-fed mice, only exercise intervention or leucine intervention could not reduce insulin resistance induced by HFD. However, the combination of exercise and leucine might reduce systemic insulin resistance induced by HFD. Moreover, exercise and leucine intervention increased the expression of adiponectin in the adipose tissue and decreased the inflammatory response in the liver and adipose tissue during HFD feeding.

Many experiments have proved that HFD can lead to metabolic changes of body composition in mice (2–5), such as visceral fat gain, weight gain, and insulin resistance. Obese children and adolescents may be more closely related because this is the plastic period of life (4). The intervention for early obesity has a great significance for the control of diabetes or the further development of obesity. It is worth noting that exercise did not reduce the weight gain caused by HFD. The reason is that only moderate exercise intensity is not an effective strategy to lose weight induced by over-nutrition (24). Exercise and restricting calorie intake at the same time can effectively reduce body weight (25). A study found that a moderate-intensity exercise training program did not cause weight loss in HFD-fed group and did not have significant changes in insulin sensitivity and liver inflammation but decreased liver steatosis caused by HFD (31). However, in our study, we did not find exercise only can reduce liver steatosis. Considering the limited change of 4-week exercise time, the impact of relatively long exercise time on the body should be considered, which is the study we are planning to conduct in the future. In Marques’ study (9), 16 weeks of moderate intensity exercise can improve the lipid status to promote the reduction of weight and obesity (10). Exercise combined with long-term LFD diet was found to reduce the weight of inguinal fat and liver inflammation significantly. Victoria J. Vieira (33) et al. found that exercise reduced the inflammation of the whole body, and the intervention measured as long as 12 weeks was better than 6 weeks. In our study, 4 weeks exercise alone did not reduce HFD-induced adiposity, inflammation, and insulin resistance. It is suggested that short-term exercise is not enough to ameliorate HFD-induced phenotype.

However, 4 weeks exercise alone was enough to increase insulin sensitivity in LFD-fed mice. We found that after exercise intervention, fat deposition in fat cells decreased and was accompanied by increased phosphorylation of AMPK. AMPK activation was reported to phosphorylate and inhibit ACC, which is a rate limiting enzyme in fatty acid synthesis (11, 30). It is reported that exercise activates AMPK (35) and reduces ACC activity through AMPK/ACC pathway, thereby reducing fatty acid synthesis (36) and increasing fatty acid oxidation (37). Therefore, in our study, exercise might reduce adiposity by AMPK/ACC signaling pathway in the adipose tissue of LFD-fed mice. Glucose is the fuel of the human body, and there is a direct relationship between GLUT4 content in the muscle and glucose uptake ability of muscle cells (34). Studies showed that muscle glucose uptake and exercise tolerance of mice with muscle-specific GLUT4 deficiency were significantly reduced (38). Exercise training is the most powerful stimulation to increase GLUT4 expression in the skeletal muscle. After training, this effect may partly help to improve insulin function and glucose disposal and enhance muscle glycogen storage (18–20). In our study, exercise slightly increased AMPK phosphorylation (data are not shown here) and significantly increased the expression of GLUT4 level in the muscle of LFD-fed mice. Actually, ATP produced by metabolism during exercise affects the expression of AMPK (39). AMPK activation of the muscles increases rapidly during exercise (40), which is related to the duration and intensity of exercise (41). AMPK is reported to regulate GLUT4 gene expression and translocation. Exercise increases the ratio of AMP/ATP, which activates AMPK phosphorylation. AMPK phosphorylates Histone deacetylases 5 (HDAC5), which releases nuclear myocyte enhancer factor (MEF2A) to conduct the transcription of GLUT4 (38). In addition, AMPK activation promotes insulin-stimulated GLUT4 translocating from cytoplasm to membrane in muscle cells, thereby increasing glucose uptake and insulin sensitivity (42). These could be that the mechanisms of exercise increase glucose clearance in our LFD-fed mice.

Whether leucine can increase insulin resistance is still inconclusive. Some studies showed that leucine could increase insulin resistance (43, 44). The principle is that high serum leucine levels can activate mammalian target of rapamycin complex 1 (mTORC1) signaling pathway, which leads to inhibition of glucose transport in the muscle and adipose tissue (43) and further promotes insulin resistance and obesity (45). Leucine was reported to impair the beneficial effects from resveratrol in diabetic rats (39). However, other studies found that leucine would reduce insulin resistance (18, 46). Takegoshi et al. found that branched-chain amino acids such as leucine decreased hepatic steatosis and inflammation in HFD-induced non-alcoholic steatohepatitis model mice (46). Leucine was also reported to reduce HFD-induced inflammation in adipose tissue and normalize the level of adiponectin (18). We found leucine supplementation alone did not improve insulin resistance either in LFD or HFD-fed mice. However, combined leucine supplementation and exercise decreased pro-inflammatory response in the liver and adipose tissue as shown by reducing the level of phosphorylation of NF-*κ*B p65. Leucine and exercise also improved the mRNA level of adiponectin in adipose tissue of HFD-fed mice. In Li’s study, leucine ameliorated hepatic level of triglycerides induced by HFD in rats (47). We also found that compared with LFD group, the mRNA expression of FAS and ACC in the liver of HFD-fed mice increased by two times and one time respectively, while in leucine treated HFD-fed mice, the mRNA levels of FAS and ACC had the trend of reduction, but not significant (data are not shown here). These results suggest that leucine may also achieve this change by reducing the expression of adipogenic genes in the liver.

We found that dietary supplementation with leucine or/and physical exercise did not significantly change HFD-induced body weight and adiposity in a short time. Considering the future study, the intervention time can be extended appropriately. However, after only 4 weeks of intervention in LFD-fed mice, it can be seen that exercise significantly increased the systemic insulin sensitivity by increasing GLUT4 expression in the muscle and decreasing adiposity through increasing AMPK phosphorylation in the adipose tissue. Exercise and leucine intervention might reduce insulin resistance induced by HFD, which was associated with decreased liver and adipose tissue inflammation. The level of adiponectin is usually negatively correlated with insulin resistance and inflammation (40, 41, 48) and positively correlated with weight loss and/or exercise (35–37). In this study, adiponectin in HFD-fed mice group with exercise and leucine treatment increased significantly, which was consistent with the decrease of inflammation in the adipose tissue. The anti-inflammatory mechanism of exercise combined with leucine may need further study, but according to the existing results, we find that short-term exercise or/and leucine intervention have different mechanisms between mice fed with LFD and HFD.

## Data Availability Statement

The raw data supporting the conclusions of this article will be made available by the authors, without undue reservation.

## Ethics Statement

The animal study was reviewed and approved by the Institutional Animal Care and Use Committee at Shandong University.

## Author Contributions

XJ and YZ conducted most of experiments. WH, YL, and LZ participated in some animal experiments. XJ and XG wrote most of this article. BW and JZ wrote some sections. BW and XG made the final editing. XG came up with the concept. All authors contributed to the article and approved the submitted version.

## Funding

This research was funded, in whole or in part, by the Fundamental Research Funds of Shandong University, grant number 2017TB0028; Young Scholars Program of Shandong University, grant number 2018WLJH33; and National Natural Science Foundation of China, grant number 81803224.

## Conflict of Interest

The authors declare that the research was conducted in the absence of any commercial or financial relationships that could be construed as a potential conflict of interest.
