# Supplementary material for: Different Effects of Leucine Supplementation and/or Exercise on Systemic Insulin Sensitivity in Mice
Source: Front Endocrinol (Lausanne). 2021 May 12;12:651303. doi: 10.3389/fendo.2021.651303 (PMC8150005; doi:10.3389/fendo.2021.651303)
Supplement: Supplementary file 1 [file DataSheet_1.pdf]

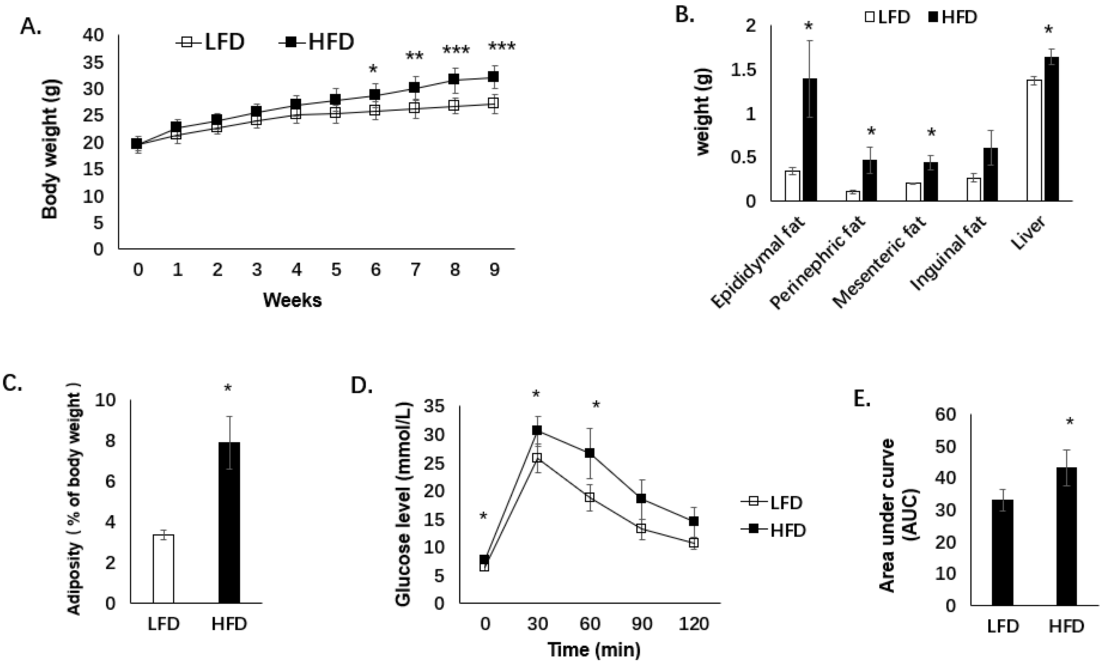

Figure S1. HFD increased body weight, fat content, liver weight, and glucose intolerance. C57BL/6J male mice of 5-6 weeks were fed with LFD and HFD respectively for 9 weeks. (A) Body weight changed from 0 to 9 weeks. (B) Weight of adipose tissue and liver in mice fed with LFD and HFD. (C) Adiposity of mice fed with LFD and HFD. Calculate the percentage of total fat content in body weight. (D) Glucose tolerance test. Mice were fasting overnight, D-glucose (2g/kg) was injected intraperitoneally. Blood glucose concentration was detected 0 minutes before injection and 30, 60, 90, and 120 minutes after injection. (E) Area under curve of GTT. The data are mean±s.d. (error bars), for A, D and E, n=30 mice per group; for B and C, n=6 mice per group. *, $p < 0.05$; **, $p < 0.01$； ***, $p < 0.001$. LFD versus HFD.

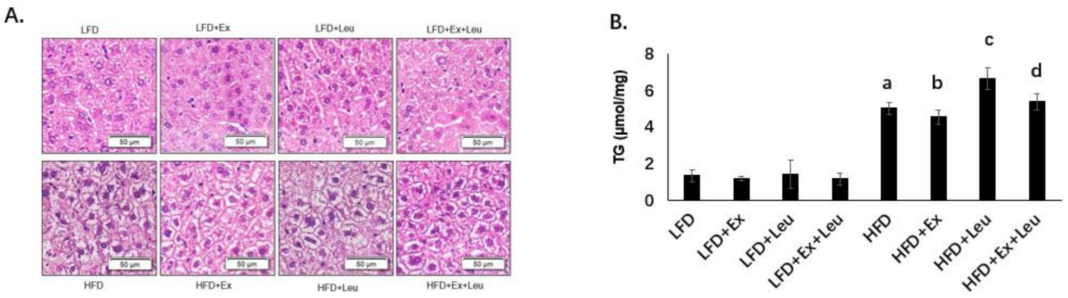

Figure S2. The effects of exercise or/and leucine on fat accumulation in liver under LFD or HFD feeding. C57BL/6J male mice of 5-6 weeks were fed with LFD or HFD. Leucine with a concentration of 1.5% and/or moderate intensity exercise was added in the 10th week and administrated for 4 weeks. (A) Liver histology. The sections of liver were stained with hematoxylin and eosin. (B) Triglycerides (TG) level in liver. TG level was normalized with protein concentration. The data are mean±s.d. (error bars), n=6 mice per group. a, $p < 0.001$, LFD versus HFD; b, $p < 0.01$, LFD+Ex versus HFD+Ex; c, $p < 0.01$, LFD+Leu versus HFD+Leu; d, $p < 0.001$, LFD+Ex+Leu versus HFD+Ex+Leu.

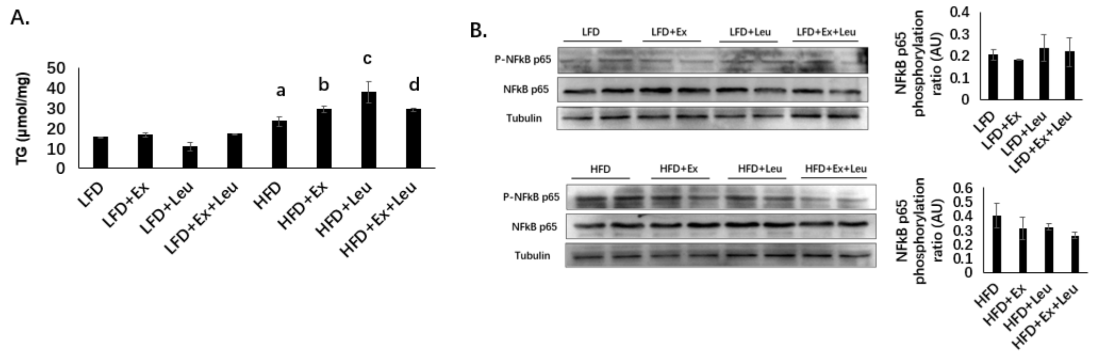

Figure S3. The effects of exercise or/and leucine on fat accumulation and inflammation in muscle under LFD or HFD feeding. C57BL/6J male mice of 5-6 weeks were fed with LFD or HFD. Leucine with a concentration of 1.5% and/or moderate intensity exercise was added in the 10th week and administrated for 4 weeks. (A) Triglycerides (TG) level in muscle. TG level was normalized with protein concentration. (B) The levels and phosphorylation states of NFkB p65 were determined using western blots. The data are mean±s.d. (error bars), n=6 mice per group. a, $p < 0.05$, LFD versus HFD; b, $p < 0.01$, LFD+Ex versus HFD+Ex; c, $p < 0.01$, LFD+Leu versus HFD+Leu; d, $p < 0.01$, LFD+Ex+Leu versus HFD+Ex+Leu.
